# Intramuscular Immunization with a Liposomal Multi-Epitope Chimeric Protein Induces Strong Cellular Immune Responses against Visceral Leishmaniasis

**DOI:** 10.3390/vaccines11081384

**Published:** 2023-08-19

**Authors:** Maria Agallou, Maritsa Margaroni, Evdokia Karagouni

**Affiliations:** Immunology of Infection Group, Department of Microbiology, Hellenic Pasteur Institute, 115 21 Athens, Greece; mariaagallou@pasteur.gr (M.A.); mmargaroni@pasteur.gr (M.M.)

**Keywords:** liposomal vaccine, multiepitope protein, visceral leishmaniasis, protection, cellular immune responses, multifunctional memory T cells

## Abstract

Control of the intracellular parasite *Leishmania* (*L*.) requires the activation of strong type 1 cellular immune responses. Towards this goal, in the present study, a multiepitope chimeric protein named LiChimera was encapsulated into cationic liposomes and its protective efficacy against experimental visceral leishmaniasis was investigated. Liposomal LiChimera conferred significant protection against *L. infantum* as evidenced by the significantly reduced parasite loads in the spleen and liver. Protection detected in Lipo:LiChimera-immunized mice was dependent on the differentiation of long-lasting cellular immune responses and particularly the induction of antigen-specific multifunctional memory CD4^+^ T_H_1 and CD8^+^ T cells that persisted during infection, as evidenced by the persistent high production of IFN-γ and IL-2 and proliferation activity. Notably, protected mice were also characterized by significantly low numbers of non-regulatory CD4^+^ T cells able to co-produce IFN-γ and IL-10, an important population for disease establishment, as compared to non-immunized control group. Collectively, these results demonstrate that cationic liposomes containing LiChimera can be considered an effective candidate vaccine against visceral leishmaniasis.

## 1. Introduction

The protozoan parasite of the genus *Leishmania* is responsible for the elicitation of leishmaniasis, a complex disease with several forms ranging from self-healing cutaneous lesions to visceral leishmaniasis (VL). VL, caused by *Leishmania* (L.) *donovani* and *L*. *infantum* is the most severe form of disease with high rates of morbidity and mortality [1]. To date, VL preventive strategies have limited themselves to vector and reservoir control as well as treatment in endemic areas. As the vector develops insecticide resistance and the parasite exhibits signs of drug resistance, the development of a prophylactic vaccine against VL to eradicate infection is strongly considered [2]. While, in cases of zoonotic VL, LeishTec^®^, Leishmune^®^, and LetiFend^®^ are the three currently available licensed commercial vaccines in Brazil and Europe, respectively [3,4]; there is no effective long-lasting vaccine in routine use against any form of the human disease except for the use of live virulent parasite vaccines [5]. However, due to adverse reactions and safety issues this immunization protocol has been abandoned.

VL establishment is associated with impaired T_H_1 responses characterized by down-regulation of IL-12 and IFN-γ along with up-regulation of T_H_2 cytokines, such as IL-4 and IL-10 [6,7,8]. Moreover, in leishmaniasis antibodies are ineffective since the parasites are rapidly taken up by the phagocytes and are, thus, inaccessible to antibodies due to their intracellular existence [8]. Therefore, immunity against leishmaniasis and eventually ideal vaccine development depends on cellular immune responses involving the development of both memory CD4^+^ T_H_1 cells and cytotoxic CD8^+^ T cells along with innate immune cells, based on studies in animals and humans [9,10,11,12]. 

In general, the design of vaccines that require the induction of T cell-mediated immunity is usually different than vaccines that target the production of neutralizing antibodies. Subunit vaccines consisted of multiple T cell epitopes obtained from different proteins are good alternatives to the use of individual protein vaccines, since T cells from genetically distinct populations recognize and respond to specific peptide epitopes [13]. However, these kind of vaccines are less immunogenic and, therefore, require an adjuvant to elicit strong immune responses [14,15,16,17,18]. Among different vaccine carriers, liposomes are considered a primary choice of safe and effective human-compatible antigen-delivery vehicles, as they are non-toxic and have inherent efficacy to induce cell-mediated responses against poorly immunogenic proteins or peptide antigens [19,20]. Moreover, they facilitate antigen uptake and presentation into the MHCII pathway of phagocytic antigen-presenting cells (APCs), including macrophages and dendritic cells (DCs) that are responsible for the induction of antigen-specific T cell responses. Importantly, cationic or positively charged liposomes, such as dimethyl-dioctadecylammonium bromide (DDAB) liposomes, are preferentially used for vaccine development against different viruses and bacteria as well as tumors [21,22,23,24,25]. This is based on their ability to encapsulate antigens, prolong antigen deposition at the injection site and enhance antigen internalization by APCs, as compared to their neutral and anionic counterparts, due to the interaction of cationic surface charge with negatively charged cell membrane [26,27]. As a result, cationic liposomes facilitate antigen’s cross-presentation to MHCI pathway and, hence, they efficiently induce CD8^+^ T cell response [28]. Importantly, previous studies have shown that soluble *Leishmania* antigen as well as specific parasite proteins formulated with cationic liposomes conferred almost complete protection against *L*. *donovani* challenge infection [29,30,31,32]. 

Based on the above knowledge, our group designed a multiepitope chimeric protein named LiChimera consisting of specific linear HTL and CTL epitopes obtained from *L*. *infantum* cyclophilin 2, cyclophilin 40, enolase, mitochondrial chaperonin HSP60, dihydrolipoamide dehydrogenase, and a hypothetical protein amino acid sequences that were identified via an immunoproteomic approach based on their exclusive recognition by asymptomatic host’s antibodies. Moreover, those proteins were selected as potential vaccine antigens due to their high content in MHCI and MHCII epitopes as evidenced by applying various bioinformatics tools [33]. Importantly, some of these proteins were shown to play a role in parasite viability and resistance against the treatment of disease [34,35,36,37,38]. Additionally, enolase, cyclophilins, and chaperonin HSP60 have been identified in the secretome of *Leishmania* species in various proteomics studies [39,40,41], which is the constituent of commercially available vaccine, CaniLeish^®^ [42]. Regarding, enolase and cyclophilins, various studies have also proven their capacity to induce protective T_H_1 immune responses in the experimental models of VL when used as a vaccine [43,44,45,46]. Subsequently, the chosen epitopes were linked together with appropriate linkers as well as with the amino-terminal end of *Mycobacterium tuberculosis* heparin-binding hemagglutinin (HBHA) serving as a built-in adjuvant [47]. LiChimera was initially tested in formulation with Addavax, a synthetic analog of MF59 (squalene-based oil-in-water adjuvant) in *Leishmania*-susceptible BALB/c mice. We reported that the immunization of mice with Lichimera adjuvanted with Addavax conferred significant protective efficacy against experimental VL through the elicitation of high antigen-specific antibody titers and CD8^+^ T cell responses. On the contrary, LiChimera alone failed to elicit long-term immune responses and subsequently did not confer protection against infection [47]. 

Considering the importance of adaptive cellular immunity in the control of *Leishmania* infection, we wanted to construct a more efficacious vaccine that will be able to induce combined strong CD4^+^ and CD8^+^ T cell responses. Thus, in the present study, we encapsulated LiChimera into DDAB:CHOL:OA cationic liposomes and we investigated its protective potential against experimental VL using the BALB/c mouse model. Cationic DDAB liposomes that were used in the present study, were further improved in terms of stability and membrane free volume by adding cholesterol (CHOL) and oleic acid (OA). As a result, we have shown that they are relatively inert in terms of activating immune responses and are non-toxic, which are important characteristics in vaccine development [48]. Additionally, we were able to show that immunization with liposomal LiChimera elicited the recruitment mainly of DCs in respect to the total number of other antigen-presenting cells (APCs) in draining lymph nodes, as compared to liposome controls, leading to antigen-specific CD4^+^ and CD8^+^ T cell differentiation and proving their excellent potency not only as antigen carriers but also as an adjuvant [48]. Accordingly, in the present study we found that liposomal LiChimera was highly immunogenic since it was able to elicit antigen-specific T_H_1-type CD4^+^ and CD8^+^ T cells in the spleen that contributed to the control of infection. 

## 2. Materials and Methods

### 2.1. Mice and Parasites

Female BALB/c mice aged between 6 to 8 weeks were used and reared under specific pathogen-free conditions in the animal care facility of the Hellenic Pasteur Institute (HPI). 

*L. infantum* (MHOM/GR/2001/GH8 strain) was used and maintained by passage to BALB/c mice. Parasites obtained from the spleens of infected BALB/c mice were cultured in vitro at 26 °C in complete medium consisting of RPMI-1640 (Biowest) supplemented with 10% (*v*/*v*) heat-inactivated fetal bovine serum (Biowest), 10 mM HEPES, 2 mM L-glutamine, 24 mM NaHCO3, 100 U/mL penicillin, and 10 µg/mL streptomycin. The soluble *Leishmania* antigen (SLA) was prepared from stationary phase *L. infantum* promastigotes cultures according to a previously described protocol [49].

### 2.2. Preparation and Characterization of Liposomes Containing LiChimera

LiChimera was expressed by a plasmid containing its encoding gene. The construction of the plasmid as well as protein expression and purification were commercially conducted by GeneCust (Labbx, Dudenange, Luxembourg) as described previously [47]. Empty liposomes and liposomes containing LiChimera consisting of DDAB, cholesterol (CHOL), and oleic acid (OA) at a molar ratio DDAB:CHOL:OA = 2:2:3 were prepared by using a hydration/extrusion method and characterized according to the methods described in Agallou et al. [48]. Based on physicochemical characterization the empty liposomes (Lipo) produced were of 233.8 ± 1.6 nm in size with a zeta potential of 13.8 ± 11.5 mV indicating their cationic nature. The size and the zeta potential of liposomes that had encapsulated LiChimera was increased into 457.1 ± 14.9 nm and 54.4 ± 7.7 mV, respectively, due to protein encapsulation [48].

### 2.3. Animal Immunization and Challenge Protocol

Mice were immunized twice intramuscularly (i.m.) on days 0 and 21. Vaccine formulations consisting of the chimeric protein LiChimera (10 µg/mouse) encapsulated into cationic liposomes (Lipo) (350 μg/mouse) were injected in a volume of 100 µL/mouse (50 µL/thigh), while the same dose, in terms of volume (PBS) or mass (empty liposomes), was given in mice serving as control groups. Four weeks after the boosting injection, all mice groups were intravenously challenged with 10^7^ stationary phase *L. infantum* promastigotes via the tail vein and the parasite load was estimated in the spleen and liver at 3 months post challenge.

### 2.4. Determination of Parasite Burden Using Limited Dilution Analysis (LDA)

Limiting dilution analysis (LDA) was performed in order to determine the parasite burden in the spleen and liver of infected mice. Specifically, a pre-weighted portion of liver or spleen was homogenized in Schneider’s Insect Medium and resuspended in the same medium supplemented with 20% FBS to obtain a final concentration of 1 mg/mL. Then, 2 fold serial dilutions of the tissue homogenates were conducted in 96-well cell culture plates and incubated at 26 °C, for 7 days. The presence of viable and motile promastigotes was examined after a period of 7 days. Parasite concentration per milligram of tissue was the reciprocal of the highest dilution that was positive for parasites.

### 2.5. Evaluation of Parasite-Specific Immune Responses

Splenocytes from immunized and non-immunized mice were collected at 3 months post challenge and homogenized into single-cell suspensions. Then, cells were plated at a density of 1 × 10^6^ cells/well in a 24-well flat-bottom plate and stimulated with SLA (12.5 µg/mL) for 18 h at 37 °C. Two hours before harvesting, cells were treated with Brefeldin A (10 µg/mL). Then, cells were stained for CD4 surface molecules for 20 min on ice in the dark, permeabilized with 0.1% saponin, and were then intracellularly stained with antibodies against IFN-γ and IL-10. The stained cells were acquired using a FACSCalibur flow cytometer (BD Biosciences) and analyzed using FlowJo software version 10.0 (Tree Star Inc., Ashland, OR, USA). All fluorescence-labeled anti-mouse antibodies used in flow cytometry were obtained from Biolegend: PerCP/Cyanine5.5 CD4 (Clone: RM4-5), PE IFN-γ (Clone: XMG1.2) and FITC IL-10 (Clone: JES5-16E3). Parasite-specific cytokine-producing T cells (%) were determined after subtraction of the cytokine-producing T cells (%) in medium alone from the cytokine-producing T cells (%) in medium with SLA. Samples that were not above the background for positive cytokine response were set to zero.

### 2.6. Antigen-Specific Proliferation Assay

Spleen cells were isolated from immunized and non-immunized mice at 3 months post challenge, as described in the above paragraph, for the evaluation of antigen-specific proliferation. For this purpose, cells were cultured in 96-well round-bottom plates (2 × 10^5^ cells/200 µL/well) in the presence of LiChimera (2.5 µg/mL) for 96 h, at 37 °C, and 5% CO_2_. Spleen cells stimulated with concanavalin A (ConA; 6 µg/mL) served as a positive proliferation control, whereas spleen cells in medium alone were used as the negative control. The cell proliferation was measured by measuring [^3^H]-TdR (Perkin Elmer, Boston, MA, USA) incorporation during the last 18 h of culture with the help of a β-counter (Microbeta Trilux, Wallac, Turcu, Finland). Proliferation was obtained as counts per minute (cpm) and the results were presented as a Stimulation index (S.I.) based on the formula below:

S.I. = cpm in the presence of antigen or mitogen/cpm in medium alone.

### 2.7. Evaluation of Antigen-Specific Cytokine and NO Production

Spleens were collected 10 days after the final immunization and 3 months post challenge and homogenized into single cell suspension. Splenocytes were seeded in 24-well plates at a density of 2 × 10^6^/well and stimulated with LiChimera (5 µg/mL) for 72 h or SLA (12.5 µg/mL) for 48 h at 37 °C for the detection of cytokine secretion and NO, respectively, in cell-free culture supernatants. IL-2, IL-4, IL-10, GM-CSF, IFN-γ, and TNFα production was assayed using Bio-Plex Pro™ Mouse Cytokine Standard, Group I kit according to manufacturer’s manual. NO production was estimated via the Griess reaction. Specifically, an amount of supernatant (50 µL) was mixed with an equal volume of Griess reagent and incubated for 10 min at room temperature. The Griess reagent consisted of 1% sulfanilamide and 0.1% N-1-naphthylethyleme diamine hydrocholide in 50% H_3_PO_4_. Then, the absorbance was measured using a spectrophotometer at 570 nm. The results were expressed in µM.

### 2.8. Detection of Antigen-Specific Memory and Multifunctional T cells

Splenocytes obtained at 10 days post boosting and 3 months post challenge were collected, plated at a density of 1 × 10^6^ cells/well in a 24-well flat-bottom plate, and stimulated with LiChimera (5 µg/mL) for 18 h at 37 °C. The cells were collected and stained with antibodies against CD4, CD8, CD44, and CD62L to assess the memory phenotype of T cells. For intracellular cytokine staining, splenocytes were collected at 10 days after second immunization and stimulated with LiChimera (5 µg/mL) for 6 h. During the final 4 h of incubation 10 µg/mL of brefeldin A (Cayman, MI, USA) were added to each well. Then, cells were stained for CD4 or CD8 surface molecules, and IL-2, IFN-γ and TNFα as described in paragraph 2.5. The stained cells were acquired using a FACSCalibur flow cytometer and analyzed using FlowJo software version 10.0. All fluorescence-labeled anti-mouse antibodies used in flow cytometry were obtained from Biolegend: PerCP/Cyanine5.5 CD4 (Clone: RM4-5), PerCP/Cyanine5.5 CD8a (Clone: 53–6.7), APC CD44 (Clone: IM7), PE CD62L (Clone: MEL-14), APC IL-2 (Clone: JES6-5H4), PE IFN-γ (Clone: XMG1.2) and FITC TNFα (Clone: MP6-XT22). The Ag-specific cytokine-producing T cells (%) were determined by subtracting the cytokine-producing T cell (%) in medium alone from the cytokine-producing T cells (%) in medium with the antigen. Samples that were not above the background for positive cytokine response were set to zero.

### 2.9. Statistical Analysis 

All data were included in the analyses, and no outliers were excluded in calculations of means/statistical significance. In the animal studies, the sample size with adequate statistical power was determined based on free-to use G*Power software (https://www.psychologie.hhu.de/arbeitsgruppen/allgemeine-psychologie-und-arbeitspsychologie/gpower accessed on 25 June). The number of samples are indicated on each figure legend. The difference between groups was determined by applying one-way ANOVA or two-way ANOVA with a multiple comparisons Tukey–Kramer post hoc test. All analyses were performed using GraphPad Prism version 9.5.0 software (San Diego, CA, USA). The results were presented as the mean ± SEM and the values with *p* < 0.05 were considered significant for all analyses.

## 3. Results

### 3.1. LiChimera Encapsulated in Liposomes Protects against L. infantum Challenge

**Figure 1:**
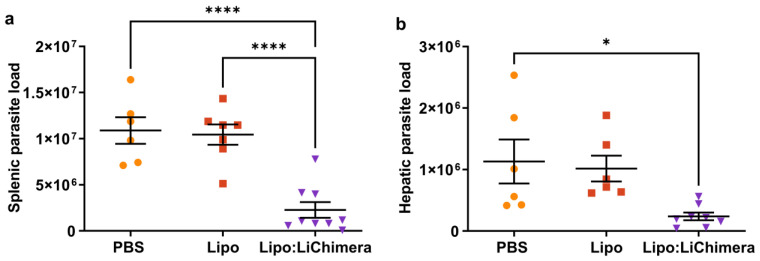
Immunization with Lipo:LiChimera provided significant protection against *L. infantum* challenge. BALB/c mice (n = 6–8) were intramuscularly immunized twice at a 3-week interval with Lipo:LiChimera (triangle). Non-immunized mice (PBS; circle) or mice immunized with empty liposomes (Lipo; square) served as control groups. One month after the second immunization, mice were intravenously challenged with 10^7^ *L. infantum* promastigotes. At three months post challenge, the parasite load in the spleen (**a**) and liver (**b**) was assessed using limiting dilution assay. Statistics were performed using a one-way ANOVA followed by a Tukey–Kramer multiple-comparisons test. The results are presented as the mean ± SEM. * *p* < 0.05, **** *p* < 0.0001

In an effort to develop an experimental vaccine candidate against VL that is able to direct cell-mediated immune responses against intracellular parasite *Leishmania*, we previously designed a multiepitope chimeric protein named LiChimera and encapsulated it into cationic liposomes (Lipo:LiChimera). As vaccine construct, in the previous study of ours Lipo:LiChimera exhibited excellent capacity of inducing dendritic cell maturation and activation in draining lymph nodes after intramuscular injection, and eventually stimulated the induction of antigen-specific CD4^+^ and CD8^+^ T cell responses [48]. Subsequently, to determine the protective efficacy of Lipo:LiChimera against *L. infantum*, BALB/c mice were immunized intramuscularly twice at 20-day intervals and 1 month later were intravenously challenged with *L. infantum* promastigotes. Protective efficacy was determined at 3 months post challenge corresponding to the chronic phase of disease via parasite load assessment in the spleen and liver in non-immunized and immunized challenged mice. Parasite load determination in the spleen and liver at this time point revealed that in Lipo:LiChimera-immunized mice the splenic parasite load was reduced by 90% and hepatic parasite load was inhibited by 80% at 3 months post challenge compared with the non-immunized (PBS) and empty liposome-immunized (Lipo) mice groups (Figure 1). Importantly, parasite load reduction in the spleen was followed by reduced splenomegaly in Lipo:LiChimera-immunized mice (Figure S1). 

### 3.2. Lipo:LiChimera Immunization Protects against L. infantum Challenge via Maintenance of Antigen-Specific T_H_1-Biased Immune Response

**Figure 2:**
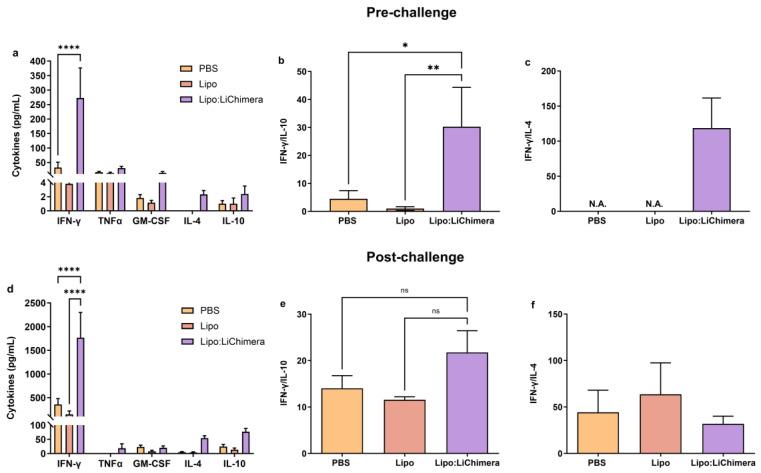
Immunization with Lipo:LiChimera induces a long-lasting antigen-specific T_H_1-type over T_H_2-type immune response. BALB/c mice (n = 6–8) were intramuscularly immunized twice at a 3-week interval with Lipo:LiChimera. Non-immunized mice (PBS) or mice immunized with empty liposomes (Lipo) served as control groups. One month after the second immunization, mice were intravenously challenged with 10^7^ *L. infantum* promastigotes. Ten days post second immunization (**a**–**c**) and 3 months post challenge (**d**–**f**), spleen cells were isolated and in vitro stimulated with LiChimera. IFN-γ, TNFα, GM-CSF, IL-4, and IL-10 levels were detected in culture supernatants after 72 h of incubation (**a**,**d**). IFN-γ/IL-10 and IFN-γ/IL-4 ratios at 10 days post immunization (pre-challenge, (**b**,**c**)) and 3 months post challenge (**e**,**f**). Statistics were performed using two-way and one-way ANOVA followed by a Tukey–Kramer multiple-comparisons test. The results are presented as the mean ± SEM. * *p* < 0.05, ** *p* < 0.01, **** *p* < 0.0001, ns: not significant, N.A.: not applicable.

To fully characterize the nature of the immune responses raised in Lipo:LiChimera-immunized mice, we analyzed the antigen-specific cytokine secretion profile in all three mouse groups prior and post challenge with *L*. *infantum*. To assess these responses, isolated splenocytes were in vitro restimulated with LiChimera and the levels of T_H_1 (IFN-γ, TNFα, GM-CSF) and T_H_2 skewed cytokines (IL-4 and IL-10) were measured. According to the results, elevated levels of IFN-γ (Lipo:LiChimera: 272.5 ± 103.7 pg/mL vs. PBS: 32.67 ± 18.34 pg/mL and Lipo: 2.5 ± 1.63 pg/mL, *p* < 0.0001) and GM-CSF (Lipo:LiChimera: 15.67 ± 4.46 pg/mL vs. PBS: 1.83 ± 0.48 pg/mL and Lipo: 1.17 ± 0.31 pg/mL, *p* = 0.9549) were detected in the Lipo:LiChimera immunized group compared to PBS and Lipo-immunized control groups at 10 days post immunization (Figure 2a). Regarding T_H_2 skewed cytokines, IL-10 (Lipo:LiChimera: 2.4 ± 1.12 pg/mL vs. PBS: 1.0 ± 0.45 pg/mL, *p* = 0.9981) and IL-4 (Lipo:LiChimera: 2.3 ± 0.56 pg/mL vs. PBS: 0.0 ± 0.0 pg/mL, *p* = 0.9981) exhibited a small non-significant increase in Lipo:LiChimera-immunized mice compared to the non-immunized control group. However, their levels in immunized mice were significantly lower than the levels of IFN-γ (IFN-γ: 272.5 ± 103.7 pg/mL vs. IL-10: 2.4 ± 1.12 pg/mL and IL-4: 2.3 ± 0.56 pg/mL), suggesting that immunization with Lipo:LiChimera skewed immune responses towards a T_H_1 phenotype (Figure 2a). Indeed, estimation of IFN-γ/IL-10 and IFN-γ/IL-4 ratios, which is known to be good correlates of enhanced T_H_1 activation, gave a value equal to 30 and 119, respectively, in Lipo:LiChimera-immunized mice. On the contrary, non-immunized mice gave a significantly lower ratio of IFN-γ/IL-10 equal to 4.5 (*p* = 0.0227; Figure 2b), whereas IFN-γ/IL-4 it could not be estimated due to zero production of IL-4 after in vitro stimulation with LiChimera (Figure 2c). 

Importantly, Lipo:LiChimera-immunized mice continued to produce statistically significant high amounts of IFN-γ at 3 months post challenge with *L. infantum* which were ~5 fold higher compared to both control mice groups (Lipo:LiChimera: 1767.0 ± 532.36 pg/mL vs. PBS: 356.0 ± 127.17 pg/mL and Lipo: 144.67 ± 73.14 pg/mL, *p* < 0.0001) (Figure 2d). Detection of T_H_2 cytokines indicated a ~13 fold and ~4 fold induction of IL-4 and IL-10 production, respectively, in Lipo:LiChimera-immunized mice compared to non-immunized and Lipo-immunized challenged mice, which were not significant (Figure 2d). Estimation of IFN-γ/IL-10 ratio for determination of the type of immune response showed that in Lipo:LiChimera-immunized mice the IFN-γ/IL-10 ratio continued to be higher than in the non-immunized mice (Lipo:LiChimera: 22 vs. PBS: 14; Figure 2e). In contrast, a slight reduction was found among groups regarding IFN-γ/IL-4 ratio (PBS: 44 vs. Lipo:LiChimera: 32; Figure 2f). 

### 3.3. Lipo:LiChimera-Induced Protection Correlates with Decreased Numbers of Regulatory T Cells in the Spleen 

**Figure 3:**
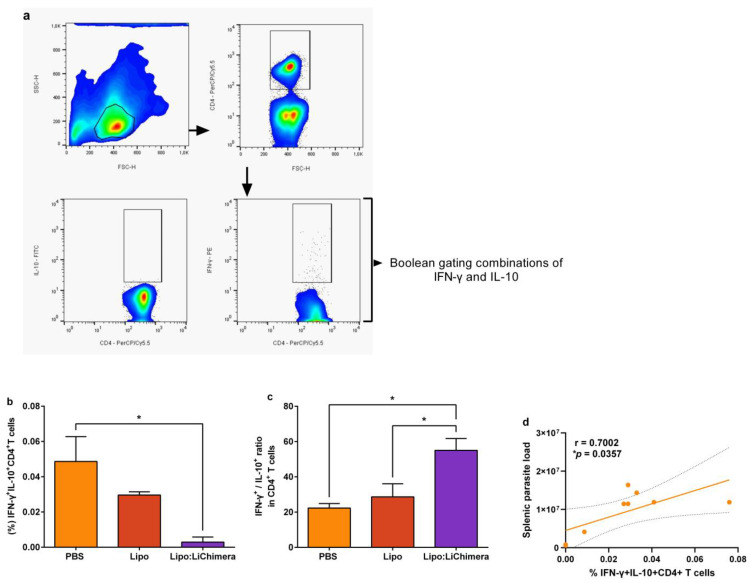
Lipo:LiChimera-immunized mice exhibited decreased numbers of IFN-γ^+^IL-10^+^ double-producing CD4^+^ T cells in infected spleens. BALB/c mice (n = 5–6) were intramuscularly immunized twice at a 3-week interval with Lipo:LiChimera. Non-immunized mice (PBS) or mice immunized with empty liposomes (Lipo) served as control groups. One month after the second immunization, mice were intravenously challenged with 10^7^ *L*. *infantum promastigotes*. Spleens were collected at 3 months post challenge for the characterization of parasite-specific CD4^+^ T cells after spleen cell in vitro stimulation with SLA for 18 h and flow cytometry. (**a**) Representative plots of the gating strategy to characterize the parasite-specific CD4^+^ T cells double- or single-producing IFN-γ and IL-10 cytokines using Boolean gating. (**b**) Frequency (%) of IFN-γ^+^IL-10^+^CD4^+^ T cells and (**c**) ratio of IFN-γ^+/^IL-10^+^ producing CD4^+^ T cells. Data are reported as the mean ± SEM. Statistics were performed using a one-way ANOVA followed by a Tukey–Kramer multiple-comparisons test. (**d**) Correlation of IFN-γ^+^IL-10^+^CD4^+^ T cell frequency (%) with parasite load in non-immunized and immunized mice. Spearman’s analysis of correlation was used to correlate the r and *p* values. The lines represent least-squares, best-fit linear regression. * *p* < 0.05.

Since establishment of VL in the spleen is attributed to the expansion of CD4^+^ T cells co-producing IFN-γ and IL-10 cytokines [50], flow cytometry analysis was conducted in order to characterize the type of parasite-specific CD4^+^ T cells at 3 months post challenge (Figure 3a). Results revealed that Lipo:LiChimera-immunized mice contained significantly lower numbers of parasite-specific IFNγ^+^IL-10^+^ double-producing CD4^+^ T cells compared to non-immunized control group (0.003 ± 0.003% vs. 0.049 ± 0.02; *p* = 0.0195) (Figure 3b). Moreover, evaluation of parasite-specific single cytokine production by CD4^+^ T cell populations was conducted in terms of IFN-γ^+^ and IL-10^+^ cells and the ratio of those two populations was estimated, since it serves as an additional correlate of protection against VL [51]. Lipo:LiChimera-immunized mice also exhibited a significant higher IFN-γ^+^/IL-10^+^ ratio of in single producing CD4^+^ T cells (equal to 55) in comparison with the non-immunized challenged group (PBS: 22.3; *p* = 0.019), further supporting a parasite-specific T_H_1 immune response dominance (Figure 3b). As expected, immunized mice with empty liposomes exhibited similar profile in terms of the IFNγ^+^IL-10^+^ double-producing CD4^+^ T cell number and IFN-γ^+^/IL-10^+^ ratio in CD4^+^ T cells compared to the non-immunized group (Figure 3a,b). To investigate whether decreased numbers of IFN-γ^+^IL-10^+^ double-producing CD4^+^ T cells were indeed associated with parasite load restriction in the spleen, a Spearman correlation analysis between those two factors was performed in all three groups. According to the analysis, increased numbers of IFN-γ^+^IL-10^+^ double-producing CD4^+^ T cells were positively correlated with parasite load in the spleen (*p* = 0.0357; Figure 3c). 

**Figure 4:**
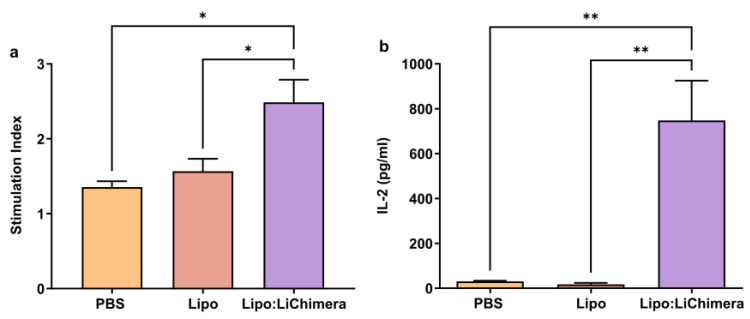
Lipo:LiChimera-immunized mice exhibited increased lymphoproliferative activity. BALB/c mice (n = 5–6) were intramuscularly immunized twice at a 3-week interval with Lipo:LiChimera. Non-immunized mice (PBS) or mice immunized with empty liposomes (Lipo) served as control groups. One month after the second immunization, mice were intravenously challenged with 10^7^ *L. infantum* promastigotes. Spleens were collected at 3 months post challenge and were in vitro stimulated with LiChimera for 96 h for the estimation of antigen-specific proliferative activity (**a**) or for 72 h for IL-2 production detection (**b**). Statistics were performed using a one-way ANOVA followed by a Tukey–Kramer multiple-comparisons test. The results are presented as the mean ± SEM. * *p* < 0.05, ** *p* < 0.01.

Importantly, the reduced numbers of IFN-γ^+^IL-10^+^ double-producing CD4^+^ T cells in the spleen were accompanied by enhanced T cell proliferation upon stimulation LiChimera in Lipo:Chimera-immunized mice compared to non-immunized (*p* = 0.0108) and Lipo-immunized (*p* = 0.0255) challenged mice, indicating a reverse of the impaired T cell responses usually found in the spleen during VL (Figure 4a). Importantly, the observed T cell proliferation was complemented with ~24 fold (PBS: *p* = 0.0062) and ~45 fold (Lipo: *p* = 0.0056) higher production of IL-2 compared to control infected mice groups (Figure 4b). 

**Figure 5:**
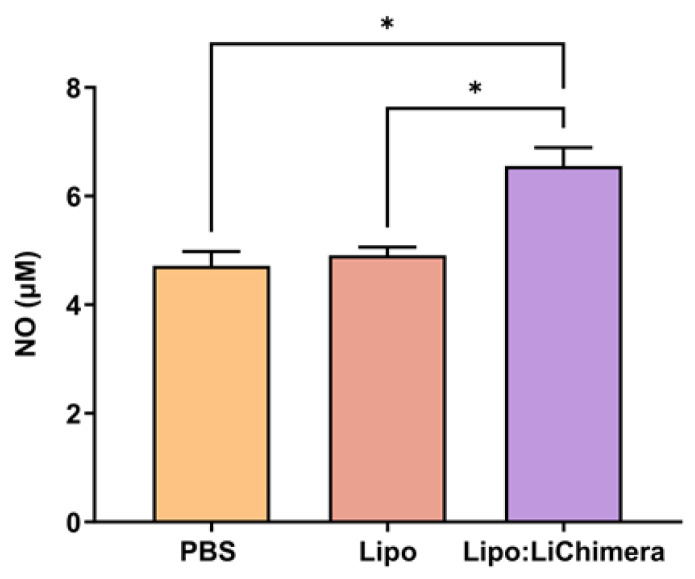
Detection of NO production at 3 months post challenge. BALB/c mice (n = 5–6) were intramuscularly immunized twice at a 3-week interval with Lipo:LiChimera. Non-immunized mice (PBS) or mice immunized with empty liposomes (Lipo) served as control groups. One month after the second immunization, mice were intravenously challenged with 10^7^ *L. infantum* promastigotes. Spleens were collected 3 months post challenge and parasite-specific NO production was estimated after a 48 h in vitro stimulation of spleen cells with SLA. Statistics were performed using a one-way ANOVA followed by a Tukey–Kramer multiple-comparisons test. The results are presented as the mean ± SEM. * *p* < 0.05.

In leishmaniasis, nitric oxide (NO) is considered a key defense mechanism against *Leishmania* parasite. Thus, nitrite production as a proxy for NO was also determined to evaluate macrophages’ leishmanicidal activity resulting from T cells activation. According to results, Lipo:LiChimera spleen cells in vitro restimulation with SLA resulted in increased amounts of NO production compared to PBS (*p* = 0.0139) and Lipo-immunized mice group (*p* = 0.014) (Figure 5), which was in accordance to the low parasite burden detected in the spleen of those mice.

### 3.4. Lipo:LiChimera Immunization Induces the Differentiation of Multifunctional CD4^+^ and CD8^+^ T Cells Immunization

**Figure 6:**
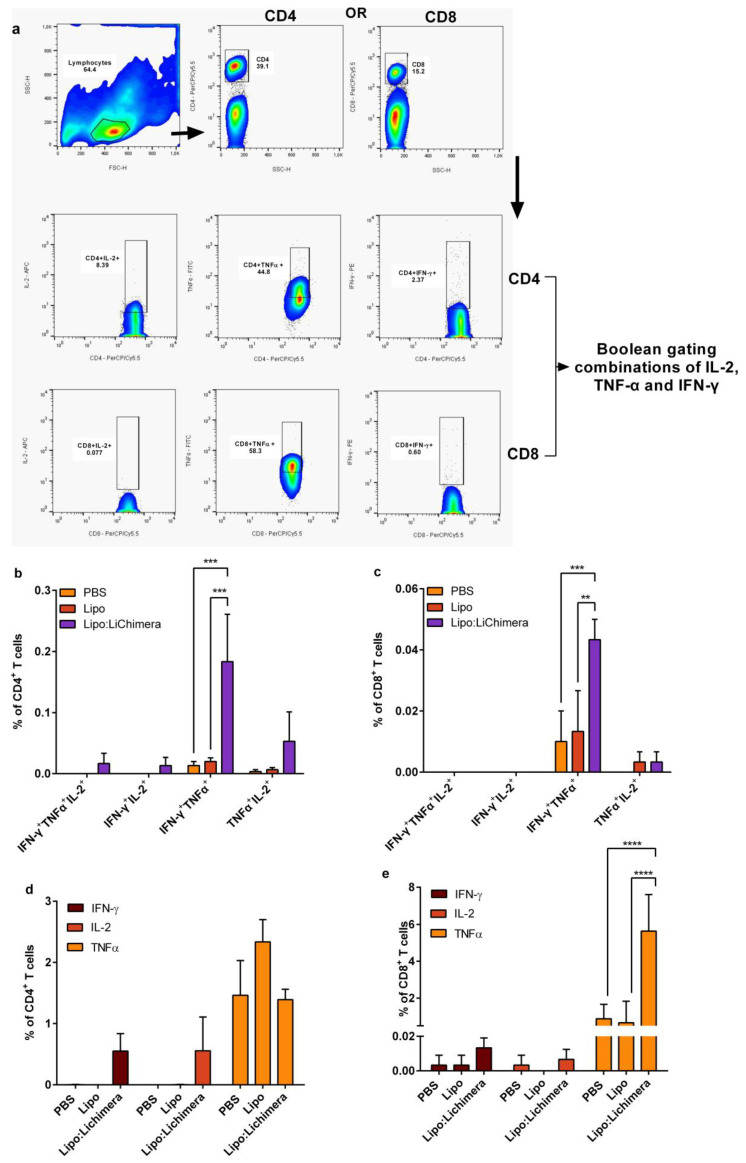
Immunization with Lipo:LiChimera induced the differentiation of antigen-specific multifunctional CD4^+^ and CD8^+^ T cells. BALB/c mice (n = 6–8) were intramuscularly immunized twice at a 3-week interval with Lipo:LiChimera. Non-immunized mice (PBS) or mice immunized with empty liposomes (Lipo) served as control groups. Ten days post immunization, spleen cells were isolated and in vitro stimulated with LiChimera for 6 h. (**a**) Representative plots of the gating strategy to characterize the LiChimera-specific multifunctional CD4^+^ T cells and CD8^+^ T cells using Boolean gating. Frequency of triple- and double-producing CD4^+^ (**b**) and CD8^+^ T cells (**c**) as well as single-producing CD4^+^ (**d**) and CD8^+^ T cells (**e**) were estimated using flow cytometry. Statistics were performed using a two-way ANOVA followed by a Tukey–Kramer multiple-comparisons test. The results are presented as the mean ± SEM. ** *p* < 0.01, *** *p* < 0.001, **** *p* < 0.001.

Subsequently, the presence of antigen-specific multifunctional CD4^+^ and CD8^+^ T cells in the spleens of the infected mice was determined. Flow cytometry analyses showed that Lipo:LiChimera-immunized mice acquired significantly high levels of double-producing IFN-γ^+^TNFα^+^ CD4^+^ T cells compared to control groups (PBS: *p* = 0.0005 and Lipo: *p* = 0.0008) (Figure 6b). Moreover, Lipo:LiChimera-immunized mice also contained high percentages of IFN-γ and IL-2 single producing CD4^+^ T cells which did not reach significance (Figure 6d), as well as increased numbers of IFN-γ^+^IL-2^+^ and TNFα^+^IL-2^+^ double producers and IFN-γ^+^TNFα^+^IL-2^+^ triple producers CD4^+^ T cells compared to control groups (Figure 6b). Among CD8^+^ T cells, although TNFα-single producing cells were dominating (5.6 ± 1.1%; Figure 6e), a significantly increased number of IFN-γ^+^TNFα^+^ double-producing CD8^+^ T cells was also found (PBS: *p* = 0.0005 and Lipo: *p* = 0.0016) (Figure 6c), although at lower levels than those detected among antigen-specific CD4^+^ T cells populations.

### 3.5. Lipo:LiChimera Immunization Induces the Differentiation of Long-Lasting Memory CD4^+^ and CD8^+^ T Cell Populations

**Figure 7:**
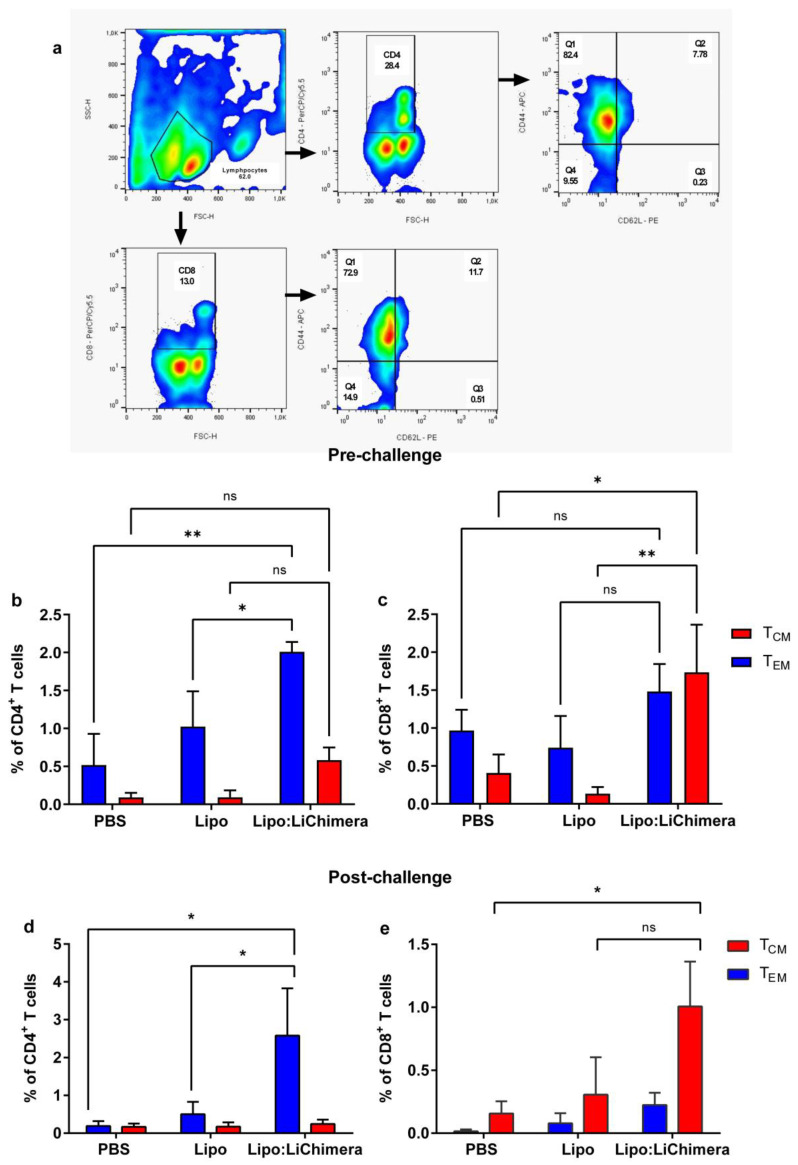
Immunization with Lipo:LiChimera induced the differentiation of antigen-specific memory CD4^+^ and CD8^+^ T cells. BALB/c mice (n = 6–8) were intramuscularly immunized twice at a 3-week interval with Lipo:LiChimera. Non-immunized mice (PBS) or mice immunized with empty liposomes (Lipo) served as control groups. One month after the second immunization, mice were intravenously challenged with 10^7^ *L. infantum* promastigotes. Ten days post second immunization (pre-challenge) and 3 months post challenge, spleen cells were isolated and in vitro stimulated with LiChimera for further analyses. (**a**) Representative plots of the gating strategy to characterize the LiChimera-specific central (T_CM_) and effector (T_EM_) memory CD4^+^ T cells and CD8^+^ T cells. T_CM_ cells were characterized by CD4^+^ or CD8^+^CD44^+^CD62L^+^ markers and T_EM_ cells were characterized by CD4^+^ or CD8^+^CD44^+^CD62L^-^ markers. Frequency of CD4^+^ (**b**,**d**) and CD8^+^ T_CM_ and T_EM_ cells (**c**,**e**) . Statistics were performed using a two-way ANOVA followed by a Tukey–Kramer multiple-comparisons test. The results are presented as the mean ± SEM. * *p* < 0.05, ** *p* < 0.01, ns: not significant.

The differentiation of memory T cells is critical for the establishment of protective immunity against infection. To determine if the Lipo:LiChimera enhanced the differentiation of the population, the phenotype of the induced antigen-specific-induced CD4^+^ and CD8^+^ T cells was assessed via applying flow cytometry analyses. We detected that Lipo:LiChimera immunization enhanced the differentiation of increased numbers of LiChimera-specific memory CD4^+^ T cells and CD8^+^ T cell populations compared to non-immunized and Lipo-immunized control mice groups (Figure 7b,c). Specifically, Lipo:LiChimera enhanced the differentiation of significant numbers of antigen-specific effector memory CD4^+^ T cells as well as central memory CD8^+^ T cell populations in the spleen compared to non-immunized and Lipo-immunized control mice (Figure 7b,c). It must be noted that, despite the fact that a 2 fold increase in central memory CD4^+^ T cells populations compared to control mice groups was detected, these populations did not reach significance (Figure 7b). Evaluation of the maintenance of antigen-specific memory T cells unveiled that Lipo:LiChimera-immunized mice increased the numbers of effector memory CD4^+^ T cells as well as central memory CD8^+^ T cells during infection compared to the numbers estimated pre-challenge with the *L. infantum* parasite (Figure 7d,e).

## 4. Discussion

Successful vaccination against *Leishmania* infection necessitates enhanced T-cell-mediated parasite clearance. In the clinical setting, vaccines enhancing the generation of both CD4^+^ and CD8^+^ T cell populations are very appealing and could be achieved by using in silico designed multiepitope antigens. This approach provides several advantages; among them the flexibility and rapidity of manufacturing process and the induction of multiple epitope-specific T cell populations [13]. In this study, we showed that homologous prime-boost immunization of BALB/c mice with a multiepitope chimeric protein named LiChimera encapsulated into cationic DDAB liposomes (Lipo:LiChimera) led to significant protection against VL without the use of immunomodulating adjuvants, further supporting that effective protein vaccines encapsulated into cationic liposomes can be successful in inducing protective immunity against diseases that require cellular immune responses. 

DCs are the most crucial APCs for initiating T cell immunity via co-stimulatory CD80/86 and CD40 expression and optimum antigen presentation to the CD8^+^ and CD4^+^ T cells via MHCI- and MHCII-increased surface expression, respectively. Accordingly, in a previous study of ours, we showed that immunization with liposomal LiChimera elicited DCs infiltration in draining lymph nodes leading to the induction of cellular immune responses, a prerequisite for achieving protection against visceral leishmaniasis [48]. In the present study, we showed that immunization with liposomal LiChimera in the absence of adjuvant resulted in significant protection against VL in the BALB/c model which was superior than that observed when given alone or as emulsion with Addavax in a previous study of ours [47]. In terms of immune correlates of protection, the LiChimera-specific cellular responses in Lipo:LiChimera-immunized mice were highly characterized by potent IFN-γ secretion and marginal levels of IL-4 and IL-10. Importantly, the high IFN-γ levels were sustained during infection while increasing production of IL-4 and IL-10 was also detected. However, the levels of both cytokines were significantly lower than those detected for IFN-γ, as evidenced by high IFN-γ/IL-4 and IFN-γ/IL-10 ratios in immunized mice group as compared to non-immunized control group. Different studies suggested that a balance between pro-inflammatory and anti-inflammatory cytokines should be achieved during immunization as well as infection in order to avoid tissue injury [52,53,54]. Thus, in our case the absence of splenomegaly and reduction of parasite load in Lipo:LiChimera-immunized mice could be the effect of balanced IFN-γ to IL-10 production due to immunization. Regarding increased LiChimera-specific IL-4 production, there are several lines of evidence supporting that not only the presence of T_H_1-type antigen-specific CD4^+^ T cells but also IL-4 production is crucial for the clearance of the *Leishmania* parasite from the liver and spleen via priming of long-term CD8^+^ T cell memory responses and eventually the induction of effective immune response against pathogens [55,56,57]. 

In the case of vaccine development against VL, several studies that used cationic liposomes or adenoviral vectors as antigen carriers showed that long-term protective responses against VL required the generation and expansion of both CD4^+^ and CD8^+^ T cells [18,32,58,59,60]. Accordingly, our results demonstrated that immunization with Lipo:LiChimera led to enhanced differentiation of both high numbers of central and effector memory CD4^+^ as well as central memory CD8^+^ T cells. Central memory T cells have an important role in the immune response, being responsible for the renewal of memory cells, while effector memory cells migrate to the infection site to help in pathogen elimination [61]. Thus, vaccine regimens that induce high numbers of circulating effector memory T cells show improved efficacy in human clinical trials. Indeed, we observed that when Lipo:LiChimera-immunized mice were challenged, the quality of the recall responses shifted towards a more effector-like signature as evidenced by the increased frequency of effector memory T cells, further supporting that this mice group could mount robust recall responses upon encountering the parasite resulting in enhanced protection.

Moreover, spleen cells of Lipo:LiChimera-immunized mice consisted of high quality antigen-specific CD4+ T cells composed of significant numbers IFN-γ^+^TNFα^+^ double producers followed by increased numbers of IFN-γ- and TNFα-single producing CD4^+^ T cells, as well as of TNFα^+^IL-2^+^ double and IFN-γ^+^TNFα^+^IL-2^+^ triple producers. In previous studies the IFN-γ^+^TNFα^+^CD4^+^ T cells have been characterized as the major effector memory population needed for protection against infections, among them *Leishmania* [53,62,63], whereas the earliest single secretion of TNFα and IL-2, followed by double TNFα^+^IL-2^+^ producers provides an antigen-specific reservoir of memory CD4^+^ T cells with effector potential [64,65,66]. Moreover, Lipo:LiChimera-immunized mice were also characterized by the differentiation of significant numbers of IFN-γ^+^TNFα^+^ double producers as well as TNFα-single producing CD8^+^ T cells. From a vaccine point of view, it was relevant to find an increase in the numbers of TNFα-producing CD4^+^ and CD8^+^ memory T cells as TNFα has important effects on macrophage killing activity through the induction of NO, as shown in our study and by others. TNFα along with supplementary effect of IFN-γ up-regulate iNOS production via the activation of the JAK-STAT signaling pathway [67,68]. 

Parallel evaluation of parasite-specific responses in the Lipo:LiChimera-immunized and infected mice group unveiled a high ratio of single-producing IFN-γ^+^ to IL-10^+^ CD4^+^ T cells, suggesting immune polarization towards a T_H_1-type immune response against the parasite. This was further enhanced by the significantly reduced numbers of parasite-specific double-producing IFN-γ^+^IL-10^+^CD4^+^ T cells. This cell population is characterized as IL-10-producing T_H_1 identified in cutaneous and VL lesions and favors disease progression inhibiting parasite elimination, since they are critical mediators of immunosuppression [69,70,71]. More importantly, double-producing IFN-γ^+^IL-10^+^CD4^+^ T cells are activated early in strong inflammatory settings usually found in VL [69,72]. Thus, the immunization-induced balanced IFN-γ to IL-10 production limiting inflammation could be a major factor for the reduced numbers of double-producing IFN-γ^+^IL-10^+^CD4^+^ T cells and eventually parasite load in the spleen and liver. Indeed, a significant positive correlation between the frequency of double-producing IFN-γ^+^IL-10^+^CD4^+^ T cells with parasite load in the spleen was found, accompanied by increased spleen cell lymphoproliferative activity and IL-2 production.

## 5. Conclusions

It is known that an effective vaccine against *Leishmania* parasite needs to contain multiple antigens or epitopes in order to induce strong and multiple T cell responses. In this context, in the present study we have incorporated a multiepitope chimeric protein named LiChimera into cationic liposomes as a candidate vaccine against leishmaniasis that fulfilled several important criteria in terms of biosafety and immunogenicity. In summary, we demonstrated that immunization with Lipo:LiChimera provided significant protection against *L. infantum* challenge via antigen-specific multifunctional CD4^+^ and CD8^+^ T cells induction leading to macrophage activation and sustained parasite-specific double-producing IFN-γ^+^IL-10^+^CD4^+^ T cells. Taken together, the present study supports that delivery of selected antigenic epitopes in the form of multiepitope chimeric protein encapsulated into cationic liposomes with inherent adjuvanticity could be the future for the design of a safe and effective vaccine against VL.

## Data Availability

Data sharing not applicable. No new data were created or analyzed in this study. Data sharing is not applicable to this article.

## Supplementary Materials

The following supporting information can be downloaded at: https://www.mdpi.com/article/10.3390/vaccines11081384/s1, Figure S1: Lipo:LiChimera-immunized mice exhibit reduced splenomegaly after *L*. *infantum* challenge.
