# Supplementary material for: Intramuscular Immunization with a Liposomal Multi-Epitope Chimeric Protein Induces Strong Cellular Immune Responses against Visceral Leishmaniasis
Source: Vaccines (Basel). 2023 Aug 19;11(8):1384. doi: 10.3390/vaccines11081384 (PMC10459177; doi:10.3390/vaccines11081384)
Supplement: Supplementary file 1 [file vaccines-11-01384-s001.zip › vaccines-2498306-supplementary.pdf]

vaccines
MDPI

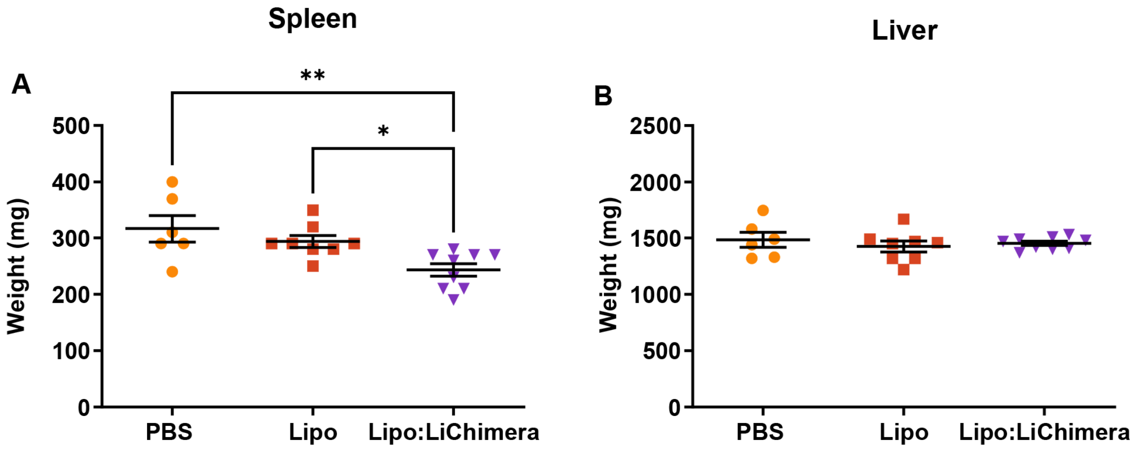

Figure S1. Lipo:LiChimera-immunized mice exhibit reduced splenomegaly after *L. infantum* challenge.
